# Acute Pancreatitis Induced by COVID-19 Vaccine: A Systematic Review

**DOI:** 10.7759/cureus.55426

**Published:** 2024-03-03

**Authors:** Akbar Hussain, Sana W Augustine, Sandhya Pyakurel, Hemika Vempalli, Rishika Dabbara, Rachel A O’dare, Ayush, Jeffrin John Varghese, Pugazhendi Inban, Malavika Jayan, Elsie Chizaram Osigwe, Sindhu Meghana Sunkara, Aadil Khan

**Affiliations:** 1 Internal Medicine, Appalachian Regional Health, Harlan, USA; 2 Internal Medicine, Liaquat University of Medical and Health Sciences, Hyderabad, PAK; 3 Internal Medicine, University of Science and Technology Chittagong, Chittagong, BGD; 4 Internal Medicine, Narayana Medical College, Nellore, IND; 5 Internal Medicine, Kamineni Institute of Medical Sciences, Hyderabad, IND; 6 Nursing, South University, Savannah, USA; 7 General Medicine, Medical University of Graz, Graz, AUT; 8 Internal Medicine, National Capital Region Institute of Medical Sciences, Meerut, IND; 9 Internal Medicine, Government Medical College & Hospital, Thiruvananthapuram, IND; 10 General Medicine, Government Medical College, Omandurar Government Estate, Chennai, IND; 11 Internal Medicine, Bangalore Medical College and Research Institute, Bangalore, IND; 12 Internal Medicine, Hayat Medical College Undergraduate, Addis Ababa, ETH; 13 Obstetrics and Gynecology, Caribbean Medical University, Willemstad, CUW; 14 Trauma Surgery, OSF Healthcare Hospital, University of Illinois College of Medicine, Peoria, USA; 15 Internal Medicine, Lala Lajpat Rai (LLR) Hospital, Kanpur, IND

**Keywords:** mumps and rubella (mmr) vaccine, measles, pancreatic acinar cells, vaccine-induced pancreatitis, covid-19, acute pancreatitis

## Abstract

Acute pancreatitis, marked by sudden inflammation of the pancreas, presents a complex spectrum of causative factors including gallstone obstruction, alcohol abuse, and viral infections. Recent studies have illuminated the emergence of vaccine-induced acute pancreatitis, notably associated with COVID-19 vaccinations, presenting diverse mechanisms ranging from direct viral-mediated injury to autoimmune reactions. Understanding this link is pivotal for public health, yet challenges persist in identifying and managing cases post-vaccination. Comprehensive literature reviews employing the PRISMA (Preferred Reporting Items for Systematic Reviews and Meta-Analyses) statement outline the potential pathways and mechanisms leading to vaccine-induced pancreatitis, emphasizing the need for deeper investigations into underlying health conditions and modifications to vaccine components. Notably, the rare occurrences of vaccine-induced pancreatitis extend beyond COVID-19 vaccines, with reports also documenting associations with measles, mumps, and rubella (MMR), human papillomavirus (HPV), and other viral vaccinations. Mechanistically, hypotheses such as molecular mimicry and immunologic injury have been proposed, necessitating ongoing vigilance and exploration. Regulatory agencies play a crucial role in monitoring and communicating vaccine safety concerns, emphasizing transparency to address potential risks and maintain public trust. Understanding and communicating these rare adverse events with transparency remain integral for informed vaccination policies and to allay concerns surrounding vaccine safety.

## Introduction and background

Introduction to acute pancreatitis and its potential relationship with vaccines

Acute pancreatitis is a prevalent condition among gastrointestinal (GI) disorders, characterized by sudden inflammation of the pancreas. The revised Atlanta classification categorizes acute pancreatitis into mild and severe forms, based on organ failure, local complications, and systemic inflammation. Mild cases involve no organ failure or complications, while severe cases feature persistent organ failure or significant local issues such as necrosis. Moderately severe cases fall between these extremes, with transient complications. Although several factors, such as gallstone obstruction of the common bile duct, alcohol abuse, and hypercalcemia, can cause this condition, the specific mechanism that initiates the condition remains unknown [1,2].

Overview of acute pancreatitis following COVID-19 vaccination

In the extensive spectrum of COVID-19 related complications, acute pancreatitis has surfaced as a rare adverse event associated with exposure to the Pfizer vaccine. A comprehensive literature review reveals mounting evidence on vaccine-induced pancreatitis encompassing mechanisms such as direct virus-mediated injury, systemic inflammatory response, circulating proinflammatory interleukins, virus-induced lipotoxicity, and drug-induced injury [2]. Notably, 8 out of 10 reports highlight the acute onset of severe abdominal pain following the first vaccine dose [3]. Further investigation indicates that the virus impacts organs expressing angiotensin-converting enzyme 2 (ACE-2) cell receptors, particularly in the GI epithelium and pancreas, specifically in the ductal and islet cells. Additional studies have demonstrated significant pancreatic injury through elevated serum amylase or lipase levels. Ozaka et al. mentioned molecular mimicry where amino acid sequences between the viral vector and self-antigens cross-react and result in autoimmune reaction against pancreatic acinar cells [4-6]. Stollberger et al. referred to other hypotheses that discuss polyclonal activation of lymphocytes, activation of self-reactive lymphocytes, and vaccine-triggered release of histamine and leukotrienes can cause tissue damage [7].

This review examines the emerging association between COVID-19 vaccination and acute pancreatitis, shedding light on potential mechanisms and clinical implications.

## Review

Brief overview of recent vaccine-induced adverse events with a special focus on COVID-19 vaccines

SARs-CoV-2 (COVID-19) vaccines have played a pivotal role in mitigating the impact of a global pandemic by demonstrating efficacy in preventing severe illnesses and death. Like all vaccines, they may elicit mild adverse effects such as pain at the injection site, fatigue, headache, muscle aches, chills, fever, and nausea. These reactions generally reflect the body’s immune response to the vaccine and are very short-lived. Despite undergoing rigorous and demonstrating safety and efficacy in larger clinical trials, increasing vaccine hesitancy persists due to safety concerns, highlighting the ongoing challenge. Although these vaccines were primarily meant for respiratory viruses, they also have some extra-pulmonary effects such as GI and neurological systems due to reported serious or even fatal adverse events [8]. Some rare and more serious adverse effects, including anaphylaxis and myocarditis or pericarditis, acute pancreatitis, Guillain-Barre syndrome, and facial palsy, have been reported [7]. It is crucial to emphasize that while these vaccines have been proven safe and effective, no vaccine is entirely without risk.

Importance of understanding vaccine-related pancreatitis for public health

Some of the limitations observed in these studies include the need for more extensive observation of cases when initial symptoms arise post-vaccination. A more detailed health history is required to assess if any underlying health conditions exacerbate or indirectly affect the condition [9]. Regarding drug-induced pancreatitis, a breakdown of the pharmacologic components triggering this pathway at a cellular level and modifications to these components can minimize adverse effects that need exploration [9]. Notably, there is a rise in pancreatitis cases among post-vaccinated individuals, and adjustments to adenoviral vector vaccines could improve safe administration to the population. Early symptom detection should be of high priority, offering a deeper understanding of the vaccine components responsible for triggering autoimmune reactions in the human body.

**Figure 1:**
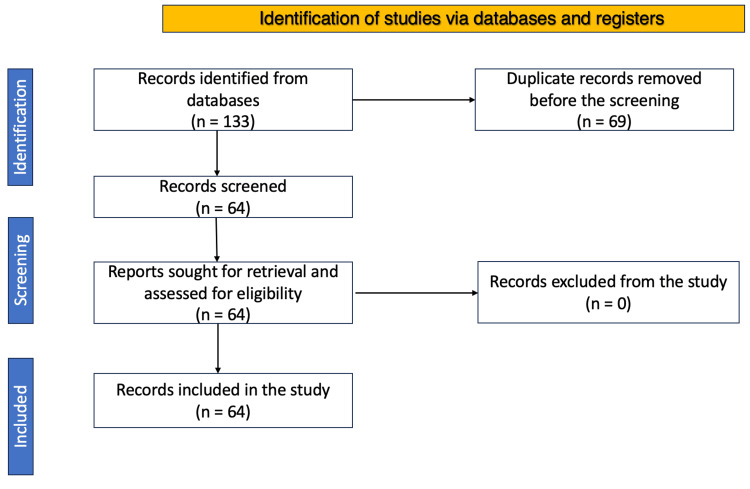
A PRISMA flow diagram PRISMA, Preferred Reporting Items for Systematic Reviews and Meta-Analyses

This review was carried out and reported in accordance with the Preferred Reporting Items for Systematic Reviews and Meta-Analyses (PRISMA) statement and standard practices in the field. The MEDLINE bibliographic database, PubMed, Google Scholar, CINAHL (Cumulative Index to Nursing and Allied Health Literature), and Scopus were searched between January 1, 2000, and December 1, 2023. Two researchers separately conducted a literature search utilizing the search method and evaluated the inclusion of papers based on their titles and abstracts. Then, the full texts of possibly admissible publications were retrieved and evaluated for inclusion. Disagreements among the researchers were resolved by debate and consensus. Later, two independent reviewers extracted the data, which was followed by a round of verification. A narrative synthesis of the findings was used to analyze the data, which required summarizing and presenting the results of the included research in a logical and intelligible manner. Initial search revealed 133 potentially relevant studies. After deleting duplicates and screening titles and abstracts, the eligibility of 64 full-text publications was evaluated, as shown in Figure 1.

Background of acute pancreatitis

Definition and Pathophysiology

Pancreatitis is an inflammatory disease of the pancreas, manifesting as severe epigastric pain, radiating to the back, along with GI And systemic symptoms such as nausea, vomiting, and fever. When this inflammation is sudden, it is termed “acute pancreatitis.” This is one of the leading culprits of hospitalization in the United States and worldwide, with its global incidence increasing year by year [10]. Irrespective of the cause, pathophysiology is nearly the same. It involves the upstream blockage of pancreatic secretion in the pancreatic duct, which prevents the exocytosis of zymogens from the pancreatic acinar cells, the exocrine unit of the pancreas. These static zymogens fuse with intracellular lysosomes to form autophagic vacuoles [11]. Cathepsin B in lysosomes stimulates the conversion of trypsinogen into its active form, trypsin. Lysosomal dysfunction and imbalance between the activators of trypsinogen (cathepsin B) and degrader of trypsin (cathepsin L) can be associated with the development of this disease [12]. Accumulation and release of trypsin in the interstitium of the pancreas leads to autodigestion of the gland. Pancreatitis occurs when the protective mechanism, which prevents abnormal activation of trypsinogen, or mechanism that reduces the activity of trypsin is overwhelmed [13].

Although pancreatic duct obstruction is an important factor, it alone is not responsible for acute pancreatitis. In a study conducted in China, it was hypothesized that pancreatic hyperstimulation in the setting of pancreatic duct obstruction leads to irregulated trypsin activity within pancreatic acinar cells, causing pancreatitis [14].

On the other hand, alcohol enhances the effects of cholecystokinin (CCK) on the pancreatic enzymes by sensitizing the acinar cells along with stimulating the release of CCK from I cells of duodenum in the small intestine [15,16]. Alcohol produces a concentration-dependent effect through cerulein, which hyperstimulates the acinar cells and activates zymogens. Cerulein, even at very low concentrations, can increase the activity of trypsin and chymotrypsin up to threefolds, and alcohol further has an array of effects ranging from disruption of phospholipid to fatty acid metabolism, affecting energy generation and disrupted intracellular signalling [17].

Common causes of acute pancreatitis

Among the many causes of pancreatitis, two stand out as the most common: bile duct obstruction by gallstones and alcoholism [18,19]. Gallstones obstruct the bile duct when they migrate downwards, subsequently leading to increased back pressure in the pancreatic duct, causing activation of pancreatic enzymes [20]. Alcohol acting through various pathways directly and indirectly cause the inflammation of the pancreas, but this correlation is not very much understood [17]. Tumors associated with the pancreatic duct is another cause of pancreatitis by the same mechanism that of gallstones. Intraductal papillary mucinous tumors bring about mechanical obstruction of the duct, causing acute inflammation by activation of the zymogens [21].

Endoscopic retrograde cholangiopancreatography (ERCP) is an iatrogenic cause of pancreatitis which, is a common adverse effect of this procedure. Probability of post-endoscopic retrograde cholangiopancreatography pancreatitis (PEP) is much more seen with procedures performed for Sphincter of Oddi Dysfunction compared with procedures performed to remove gallstones from the bile duct [22]. Other factors causing pancreatitis include drugs, hypercalcemia, and infections of viral, bacterial, and parasitic infestation [23-25].

Clinical presentation and diagnosis of acute pancreatitis

Acute pancreatitis manifests itself as severe epigastric, abdominal pain associated with nausea and vomiting. A detailed history should be taken, including any past history of pancreatitis, gallstones, and alcohol use, history of any recent procedures such as ERCP, and other biochemical parameters should be checked. A thorough physical examination will reveal signs of pancreatitis, such as jaundice, which indicates biliary obstruction [26]. Certain specific signs can be seen on inspection of the abdomen, such as Cullen’s sign and Grey Turner’s sign around the umbilicus and flanks, respectively. These are ecchymosis developed due to pancreatic necrosis [27].

Diagnosis of acute pancreatitis is made if two of the following three criteria are fulfilled: (1) severe acute epigastric pain radiating to the back, (2) serum amylase or lipase activity thrice the normal upper limit, (3) characteristic findings of acute pancreatitis on abdominal radio imaging [11].

Acute pancreatitis can be categorized into the following three categories on the basis of severity of the disease, complications, and persistent organ failure, with the help of the revised Atlanta score: (1) mild pancreatitis, which resolves within a week, (2) moderately severe pancreatitis, which is slow to resolve with delayed hospital discharge and may or may not require intervention, and (3) severe acute pancreatitis, which needs long hospital stay with requirement of intervention [28].

The most important factor in determining severity is organ failure for more than 48 hours [29]. Complications of acute pancreatitis can be local or systemic. Local complications include acute peri-pancreatic fluid collections, pseudocyst, acute necrotic collections, and walled-off necrosis, while the systemic complications are cardiovascular, renal, and pulmonary involvement either as flare-ups of the pre-existing conditions or organ failure [28].

Diagnostic modalities and treatment

For the diagnosis of pancreatitis, one can use a wide variety of modalities including biochemical markers and radio imaging.

The most commonly used biochemical markers in the diagnosis of acute pancreatitis are serum lipase and amylase. Serum lipase has a higher sensitivity compared to other serum and urine tests and remains elevated for two weeks compared to amylase levels (up to five days). Serum lipase is useful when there is a delay between onset of symptoms and point of seeking medical attention [30,31]. Ultrasound (USG) is the initial radio imaging done for all patients in whom pancreatitis is suspected. It is easily available, less expensive, has no risk of radiation exposure, and can further be used to exclude and differentiate the causes of pancreatitis ranging from gallstones to alcohol abuse [32]. Endoscopic USG, a more sensitive and modified form of classical USG, can also be used for detection of gallstones. It is a non-invasive alternative to ERCP and has an almost equal sensitivity [33]. Magnetic resonance cholangiopancreatography is another non-invasive modality mainly used to visualize pathologies related to biliary and pancreatic duct and has a high sensitivity and specificity [34]. CT scans have limited use in acute setting and are only performed to narrow down the differential diagnosis of acute pancreatitis [35].

Treatment of acute pancreatitis

Supportive Care

All the patients of pancreatitis should be kept nil per oral (NPO) and should be given oxygen, intravenous fluid resuscitation, analgesics, and prophylactic antibiotics (if required) as initial treatment. Fluid resuscitation Immediate intravascular fluid resuscitation within 24 hours at a rate of 5-10 mL/kg/hour is recommended in acute pancreatitis to prevent tissue hypoperfusion secondary to inflammation and lower the rate of systemic inflammatory response syndrome and organ failure [36-38]. Oxygen saturation (SpO2) of 98-94% should be maintained with regular monitoring. If initial saturation is below 85%, oxygen should be delivered at a rate of 15 L/min via a reservoir mask till the patient’s condition stabilizes [39]. Pain management as the disease is associated with severe abdominal pain, and administration of analgesics such as opioids or nonsteroidal anti-inflammatory drugs is of great importance, prioritizing an effective response and early relief of pain [40]. Study shows that among the various analgesics, opioids are a good choice, if not better, for pancreatitis-associated abdominal pain [41]. Nutrition acute pancreatitis is a hypermetabolic state with increased catabolism and is further worsened by nutrient sufficiency and infection [42]. Oral and enteral feeding is recommended at the earliest as it helps with reducing proinflammability response [43].

Antibiotics and Surgical Treatment

There is no specific drug recommended for acute pancreatitis treatment. For mild and moderate pancreatitis, prophylactic antibiotics are not advised. Only in the case of severe acute pancreatitis with infection, the use of antibiotics are recommended [44,45]. Antibacterial drugs having good tissue penetration, such as quinolones and metronidazole, can be used against anaerobic bacteria [44,46], whereas for gram-positive anaerobes and gram-negative bacteria, it is advisable to use piperacillin-tazobactam and third-generation cephalosporin, respectively [47]. Surgically, acute gallstone pancreatitis is treated by ERCP when it is associated with cholangitis and obstruction of the common bile duct [44].

The International Association of Pancreatology (IAP) recommends no surgery for mild acute pancreatitis. but if it is associated with the gallbladder, it is advised to perform cholecystectomy during the same hospitalization to prevent recurrence [37,48]. If necrotizing pancreatitis occurs, surgery is performed within 14 days of onset of symptoms [45], whereas walled-off pancreatic necrosis, a complication of pancreatitis occurring four weeks after onset of disease, is treated by percutaneous or endoscopic drainage, and the procedure is performed after four weeks if there are signs of organ failure, growing pseudocyst, or symptoms of gastric, biliary, or intestinal obstruction and may be extended to eight weeks of onset of disease when the symptoms are just of pain and discomfort [44]. If necrotizing pancreatitis occurs, surgery is performed within 14 days of onset of symptoms [45]. Surgical options are considered only when percutaneous or endoscopic intervention fails. Study shows that late surgery, done after four weeks of the disease onset, reduced the mortality as after four weeks the necrotic and vital tissues are clearly distinguishable and so the result of necrosectomy is better [44].

Other vaccines inducing acute pancreatitis

Acute pancreatitis has been found to be a rare complication of viral infections including measles, mumps, rubella, coxsackie B, varicella, influenza, and COVID-19. The mechanism for virus-induced pancreatitis has been hypothesized to result from swelling of the ampulla of Vater and pancreatic ducts. Another explanation based on animal models is that the virus causes a direct attack on the pancreatic acinar cell, thereby leading to acute pancreatitis [49]. There is scant reference to vaccine-induced pancreatitis in the existing literature. While exceedingly rare, there have been occasional reports of acute pancreatitis following administration of the measles, mumps, and rubella (MMR) vaccine. In a case report by Toovey and Jamieson, acute pancreatitis was diagnosed by raised serum amylase levels and due to the symptoms of pancreatitis occurring at the same time as that of parotitis. After examining the patient’s history, no other risk factors for pancreatitis, such as alcohol consumption or issues related to the gallbladder, were found [50].

A case report of a 51-year-old female highlighted her experience of developing acute pancreatitis after receiving influenza vaccinations in 2004 and 2005, following an uneventful vaccination in 2003. The potential cause, likely immunological in nature, as mentioned in the report, could explain why the patient did not experience pancreatitis after the initial vaccination [51].

Lately, the COVID-19 vaccine has been implicated in cases of pancreatitis. The exact cause of COVID-19 vaccine-induced pancreatitis remains uncertain. Similarities between vaccine components and body antigens might trigger an autoimmune response, akin to COVID-19 [52,53]. A case report by Boskabadi et al. included a healthy 28-year-old woman lacking any predisposing factors or a family history of pancreatitis. However, she experienced abdominal pain within three days following the second dose of the COVID-19 vaccine. This was later followed by limb numbness. Three months after the last vaccination, she was hospitalized due to recurrent abdominal pain, which was then diagnosed as acute pancreatitis [53]. Thus, the occurrence of acute pancreatitis following COVID-19 vaccination emphasizes the importance of continued vigilance for potential rare adverse events post-vaccination.

Mechanism and pathways

The mechanism of vaccine-induced pancreatitis is currently unknown, although there are various theories put forward to explain these rare occurrences. Heterophilic reactivity or “molecular mimicry” is the most widely accepted hypothesis. In this theory, immunologic injury may be caused by a cytotoxic antibody system that has a heterophilic reactivity to acinar cells. There are also suggestions in the literature of several additional mechanisms of immunologic injury such as poly-clonal activation (adjuvant reaction) of lymphocytes, “bystander activation” of self-reactive lymphocytes, or somatic mutation of immunoglobulin variable genes. Additional mechanisms could include secondary pancreatitis caused by vaccine-induced vasculitis or the release of anaphylactic mediators such as histamine and leukotrienes induced by vaccine antigens [54]. Bogdanos et al. demonstrated cross-reactivity between antibodies to HBsAg (hepatitis B surface antigen) and myelin basic protein (MBP) and myelin oligodendrocyte glycoprotein (MOG) after hepatitis B vaccination. This finding supports the theory of "molecular mimicry" [4]. A similar mechanism is postulated for cases of acute pancreatitis after HPV vaccination. The quadrivalent HPV vaccine is composed of virus-like particles of the L1 major capsid proteins of HPV-6, HPV-11, HPV-16, and HPV-18, which incidentally share epitopes with human proteins. Although these epitopes by themselves are unable to generate a vigorous immune response, the addition of adjuvants (aluminum) increases the antigen-specific response. Also, there are numerous reports of exacerbation of autoimmune conditions after HPV vaccination [55]. More recently, COVID-19 vaccines have been implicated in cases of post-vaccination pancreatitis [53]. Another vaccine that has been associated with post-vaccination pancreatitis is the MMR vaccine. Unlike the vaccines previously discussed, the MMR vaccine is a live vaccine capable of mounting a low-grade infection. Adler et al. described a case of pancreatitis post-MMR vaccination and hypothesized that the virus directly attacked the pancreatic acinar cells [49].

The role of regulatory agencies in monitoring and evaluating vaccine safety

Despite the overwhelming evidence of safety, vaccines are still associated with a small degree of untoward reactions, even though they are properly manufactured, handled, and administered [56]. As a result, it is an important first step in any discussion on vaccine safety to recognize that vaccines can cause undesirable side effects and that concerns associated with them are understandable [57]. Many countries currently lack the tools necessary to detect adverse events following immunization; hence, it would be imprudent to plan the introduction of vaccines into these nations until reliable procedures for tracking vaccine safety are in place. Implementing the vaccine safety monitoring system and proactive communication of safety concerns are the duties of public health authorities [58]. The prevention of allergic reactions should be adopted; this can be achieved by identifying those who are more susceptible and asking about a history of allergies to prior shots and vaccine components, which may point to an underlying hypersensitivity [59]. It is essential to have quick access to medical assistance when side effects arise to foster trust and address worries before they intensify to the point where they worsen the experience [57]. Systems in place should be able to make objective and clear communication regarding the safety issues of vaccines using a mix of communication channels that are both culturally acceptable and effective [60].

Consideration of how vaccine-related pancreatitis cases influence vaccination policies

There have been several cases of pancreatitis documented. For example, a 23-year-old male experienced acute pancreatitis following the hepatitis A vaccination [61]. A 20-year-old man, after receiving the human papillomavirus (HPV) vaccine, developed acute pancreatitis [55]. A 28-month-old was diagnosed with pancreatitis after the varicella vaccination [62]. A 28-year-old woman in good health experienced acute pancreatitis after the COVID-19 vaccination [53]. These numerous reports have proved that safety cannot be guaranteed [63]. Admitting that the safety property is relative and cannot be perfectly ensured, many modern vaccination policies now allow exemptions for people with compromised immune systems, allergies to vaccination components, or strongly held objections [64].

The importance of transparent communication with the public about potential risks

Although there are obvious short-term costs to transparent negative communication, there are no advantages to the alternative of reassuring the public about the safety and efficacy of vaccines through vague communication [65]. This approach will only serve to foster vaccine skepticism and long-term mistrust of authorities. Transparency in communication with the public has long-term benefits. It builds and sustains trust, which is a critical resource for handling both present and future health emergencies [66]. Transparency prevents conspiratorial ideas (such as the notion that political elites are dishonest and incompetent) from proliferating into hitherto untapped demographics, which would otherwise challenge health communicators trying to reach them [67]. Access to evidence-based information about vaccine adverse events, such as summaries of Vaccine Adverse Events Reporting System (VAERS) data, can help allay vaccine hesitancy worries about vaccine safety and adverse events, boost vaccine trust, and ultimately boost vaccine acceptance [68].

## Conclusions

The emergence of acute pancreatitis as a rare adverse event post-vaccination, particularly linked to COVID-19 vaccines, underscores the need for comprehensive understanding and vigilance. While vaccines play a pivotal role in mitigating diseases, their rare adverse effects, including pancreatitis, warrant attention and further exploration. Mechanisms such as molecular mimicry and immunologic injury have been proposed but necessitate deeper investigations. Regulatory agencies' roles in monitoring and transparently communicating vaccine safety concerns are pivotal for public trust and informed decision-making. Transparent communication regarding potential risks associated with vaccination remains crucial for fostering trust and ensuring informed vaccination policies. The ongoing assessment, understanding, and communication of rare adverse events such as vaccine-induced pancreatitis are essential pillars in sustaining public confidence in vaccination programs worldwide.
